# Mechanistic and Therapeutic Insights into Flavonoid-Based Inhibition of Acetylcholinesterase: Implications for Neurodegenerative Diseases

**DOI:** 10.3390/nu17010078

**Published:** 2024-12-28

**Authors:** Natalia Cichon, Weronika Grabowska, Leslaw Gorniak, Maksymilian Stela, Piotr Harmata, Michal Ceremuga, Michal Bijak

**Affiliations:** 1 Biohazard Prevention Centre, Faculty of Biology and Environmental Protection, University of Lodz, Pomorska 141/143, 90-236 Lodz, Polandleslaw.gorniak@biol.uni.lodz.pl (L.G.); maksymilian.stela@biol.uni.lodz.pl (M.S.); michal.bijak@biol.uni.lodz.pl (M.B.); 2 Faculty of Advanced Technologies and Chemistry, Military University of Technology, 2 gen. S. Kaliskiego St., 00-908 Warsaw, Poland; piotr.harmata@wat.edu.pl; 3 Military Institute of Armoured and Automotive Technology, Okuniewska 1, 05-070 Sulejówek, Poland; michal.ceremuga@witpis.eu

**Keywords:** flavanones, flavonoids, acetylcholinesterase (AChE) inhibition, cognitive enhancement, neuroprotection, neurodegenerative diseases, polyphenolic compounds, cholinergic signaling

## Abstract

Flavonoids are naturally occurring polyphenolic compounds known for their extensive range of biological activities. This review focuses on the inhibitory effects of flavonoids on acetylcholinesterase (AChE) and their potential as therapeutic agents for cognitive dysfunction. AChE, a serine hydrolase that plays a crucial role in cholinergic neurotransmission, is a key target in the treatment of cognitive impairments due to its function in acetylcholine hydrolysis. Natural polyphenolic compounds, particularly flavonoids, have demonstrated significant inhibition of AChE, positioning them as promising alternatives or adjuncts in neuropharmacology. This study specifically examines flavonoids such as quercetin, apigenin, kaempferol, and naringenin, investigating their inhibitory efficacy, binding mechanisms, and additional neuroprotective properties, including their antioxidant and anti-inflammatory effects. In vitro, in vivo, and in silico analyses reveal that these flavonoids effectively interact with both the active and peripheral anionic sites of AChE, resulting in increased acetylcholine levels and the stabilization of cholinergic signaling. Their mechanisms of action extend beyond mere enzymatic inhibition, as they also exhibit antioxidant and anti-amyloidogenic properties, thereby offering a multifaceted approach to neuroprotection. Given these findings, flavonoids hold considerable therapeutic potential as modulators of AChE, with implications for enhancing cognitive function and treating neurodegenerative diseases. Future studies should prioritize the enhancement of flavonoid bioavailability, evaluate their efficacy in clinical settings, and explore their potential synergistic effects when combined with established therapies to fully harness their potential as neurotherapeutic agents.

## 1. Introduction

Acetylcholinesterase (AChE) is a protein composed of 537 amino acids belonging to serine proteases from the serine hydrolase group. It is primarily present in neuromuscular synapses and erythrocytes, exhibiting the highest activity in the gray matter structures, i.e., the hippocampus, cerebellum, and caudate nucleus of the brain [1]. AChE activity serves to catalyze the hydrolysis of acetylcholine (ACh) into choline and acetic acid, thereby preventing continuous impulse discharge at the nerve endings. Hence, its presence is obligatory for the proper functioning of both the central and peripheral nervous system [2].

The most essential element of AChE is a 20Å-deep and 5Å-wide groove situated on the surface, extending into the molecule [2]. This groove constitutes the ligand-binding site, where substrate hydrolysis is catalyzed by the enzyme or where an inhibitor blocks its activity. The groove structure features several key functional domains, including the peripheral anionic site (PAS), located at a short distance from the groove, and the catalytically active site (CAS), situated near the groove bottom [3]. PAS plays a crucial role in the initial stages of substrate binding. It comprises amino acid residues such as tyrosine (Y124, Y337) and tryptophan (W286), which are responsible for electrostatic interactions with positively charged substrate groups, including the quaternary nitrogen atom in the ACh molecule. These interactions facilitate substrate guidance into the catalytic groove. Additionally, the tryptophan residue (W286) participates in binding lipophilic groups present in the substrate, further stabilizing its position [4]. CAS is responsible for ACh hydrolysis. The tryptophan residue plays a key role in positioning the hydrolyzed molecule by forming cation–π bonds with the quaternary nitrogen atom in ACh. The groove bottom also contains an acyl pocket, where aromatic groups from phenylalanine residues (F295, F297) are present. These residues assist in stabilizing the ACh molecule by interacting with its acetyl fragment [5].

Within CAS, there is a catalytic triad consisting of serine (S200), histidine (H440), and glutamic acid (E327) [1,6,7], which are directly involved in substrate hydrolysis. Histidine and glutamic acid enhance the nucleophilicity of serine by serving as electron donors, thereby facilitating catalysis. During catalysis, the double bond between the carbon and oxygen atoms in the ACh carbonyl group is cleaved, leading to the formation of a covalent bond between serine and the substrate. This stage results in an intermediate reaction product, where the negatively charged oxygen atom (oxyanion) is stabilized by interactions with amide groups within the oxyanion pocket [8]. In the subsequent stage of catalysis, the bond between the choline and acetyl moieties of ACh is broken, leading to the release of choline and the formation of an enzyme-bound acetylated intermediate, acetylserine (CH3CO-AChE). Subsequently, this acetyl–serine bond is hydrolyzed, with the release of acetic acid and regeneration of the active enzyme, readying it to engage in another catalytic cycle [6,7,9].

Polyphenolic compounds, also known as polyphenols, are currently among the most studied natural molecules due to their biological properties, which can be utilized in modern pharmacotherapy [10]. Numerous epidemiological studies have shown a correlation between the reduced incidence of cardiovascular diseases and cancers and the consumption of plant-based foods containing polyphenolic compounds [11,12,13]. For many years, polyphenolic compounds have been used in pharmaceutical preparations recommended for treating various liver injuries [14] and strengthening the immune system, as well as in dietary supplements used to alleviate symptoms associated with circulatory failure in the lower-limb blood vessels [15]. They are also found in soy supplements, which serve as an alternative to hormone replacement therapy aimed at preventing undesirable menopausal symptoms [16].

Epidemiological studies conducted in recent years indicate that natural organic compounds significantly not only reduce the risk of cardiovascular diseases and certain types of cancers but also exhibit strong neuroprotective effects (Table 1). These findings suggest that incorporating these compounds into dietary practices could play a critical role in enhancing overall health and preventing various diseases [17,18]. Additionally, these compounds exhibit strong inhibitory effects on enzyme activity, including serine proteases, which share structural and catalytic mechanisms of hydrolysis similar to those of AChE [19]. Consequently, there has been an increasing interest in recent years in identifying plant-derived compounds that could effectively serve as AChE inhibitors [20].

This review integrates evidence from in silico, in vitro, and in vivo studies to provide a comprehensive understanding of the mechanistic pathways through which flavonoids inhibit AChE. Unlike prior reviews, it emphasizes the dual role of flavonoids in AChE inhibition and their complementary neuroprotective mechanisms, including antioxidant, anti-inflammatory, and anti-amyloidogenic effects, highlighting their potential for multi-pathway therapeutic strategies. The aim of this study is to evaluate these mechanisms and assess the therapeutic potential of flavonoids in addressing the cognitive decline associated with Alzheimer’s disease (AD) and related neurodegenerative disorders. By focusing on specific compounds, such as quercetin, apigenin, and naringenin, this review underscores their roles in mitigating cognitive deficits, reducing neuroinflammation, and combating oxidative stress in AD models.

## 2. Inhibition of Acetylcholinesterase Activity in Pathological Conditions

The cholinergic system plays a crucial role in the proper functioning of the nervous system, and its dysregulation leads to various pathological conditions observed in neurodegenerative diseases. The current symptomatic treatment of dementia in AD is based on the cholinergic hypothesis, which attributes the primary symptoms of the disease, such as cognitive impairments (particularly memory deficits) and behavioral disturbances, to cholinergic dysfunction. In the progression of AD, disruptions in the synthesis and activity of choline acetyltransferase, as well as impairments in ACh binding to its receptors, are commonly observed [76,77]. Post-mortem studies conducted on patients with AD have revealed significant degeneration of the cholinergic neurons in the basal forebrain, particularly in the nucleus basalis of Meynert, which sends cholinergic projections throughout the cerebral cortex. Additionally, degeneration is observed in the medial septal nucleus, from which cholinergic projections are disseminated to the hippocampus [78].

Since the introduction of the first AChE inhibitors in 1997, these compounds have become the primary class of drugs used as the first-line pharmacotherapy for addressing cognitive impairments in patients with AD. At present, three AChE inhibitors—donepezil, galantamine, and rivastigmine—are clinically approved and registered for the treatment of mild to moderate stages of AD [79]. These agents function as reversible inhibitors of AChE, preventing the enzymatic hydrolysis of ACh, thereby increasing the levels of available Ach, a neurotransmitter, which is notably deficient in AD. This mechanism enhances synaptic transmission within the cholinergic system, which can temporarily improve or stabilize dementia-related symptoms [80]. Results from 22 clinical trials have demonstrated the efficacy of AChE inhibitors in treating cognitive impairments associated with AD. AChE inhibitors were found to improve concentration and attention in AD patients, likely due to a reduction in the spread of activation across memory nodes within the semantic network, which is influenced by cholinergic neurons in the frontal cortex [81]. Furthermore, a positive effect of AChE inhibitors on speech function in AD patients has also been observed [82].

Beyond their typical impact on the cholinergic system, AChE inhibitors may not only alleviate the symptoms of AD but also slow its progression by inhibiting the formation of β-amyloid plaques. Studies have shown that AChE, particularly its PAS, which is rich in aromatic amino acids, is involved in β-amyloid aggregation. A positive correlation has been observed between AChE activity and β-amyloid aggregation. PAS not only facilitates β-amyloid aggregation but also increases the neurotoxicity of amyloid fibrils [83]. Thus, AChE inhibitors, by blocking access to PAS, may inhibit amyloid aggregation and slow disease progression.

The inhibition of AChE may also be a promising therapeutic approach for other forms of dementia. Clinical trials have demonstrated that the use of AChE inhibitors has a positive effect on daily functioning and reduces behavioral disturbances in individuals with senile dementia [79].

Another condition in which AChE plays a significant role is myasthenia gravis, an autoimmune disorder in which antibodies target the ACh receptors, blocking nerve impulses to muscles, resulting in muscle weakness, tremors, and fatigue. The inhibition of AChE increases the availability of ACh, facilitating muscle fiber activation and contraction [84]. Clinical studies have shown that the use of AChE inhibitors improved muscle function in 90% of patients [85].

The cholinergic pathway also plays a crucial role in the neurohumoral anti-inflammatory mechanism. Thus, the use of AChE inhibitors may enhance cholinergic transmission and protect against inflammation within the nervous system. Studies conducted in rats have demonstrated that AChE inhibitors suppress the inflammatory response and neurodegeneration induced by bacterial lipopolysaccharides in the cortex and hippocampus [86].

AChE inhibitors also exhibit anti-inflammatory effects by acting on immunocompetent cells expressing the α7 nicotinic acetylcholine receptor, thereby inhibiting their pro-inflammatory activity. This mechanism plays a critical role in the pathogenesis of multiple sclerosis (MS), where inflammation caused by lymphocytes is responsible for demyelination and neurodegeneration [87]. The inhibition of AChE may not only prevent further demyelination but also promote the regeneration of already damaged myelin sheaths [88].

## 3. Flavonones

Polyphenolic compounds are synthesized exclusively in plant organisms as secondary metabolites, playing diverse roles in the function and protection of plants [89]. From a chemical perspective, polyphenols represent a broad group of organic compounds characterized by the presence of one or more aromatic rings, to which one or more hydroxyl groups are attached at various carbon atoms of the aromatic ring(s) [90]. Due to their significant structural diversity, polyphenols are classified into seven main groups: simple phenols, hydroxybenzoic acids, hydroxycinnamic acids, stilbenes, lignans, anthraquinones, and flavonoids [11,90,91,92].

Despite the large number of different classes of polyphenolic compounds, the flavonoid class is the most extensively studied and exhibits the highest biological activity. To date, over 7000 compounds have been characterized as members of this class, based on their chemical structure. The basic skeleton of all flavonoids consists of 15 carbon atoms and is derived from the flavone backbone, specifically 2-phenylchroman, comprising three rings: A, B, and C (general structure: C6-C3-C6) [93]. Rings A and B are aromatic, while the C ring is a pyran ring containing a three-carbon sequence, fused with benzene ring A, forming a benzopyran or pyrone structure. Although all flavonoids share this fundamental chemical structure, they exhibit considerable structural diversity, particularly in the heterocyclic C ring. Additionally, flavonoids can possess various substituents on rings A and B, such as hydroxyl, methoxyl, or isoprenyl groups. Consequently, flavonoids are further categorized into subclasses: flavanols, flavonols, flavanones, flavones, anthocyanins, and isoflavones [94,95].

**Figure 1:**
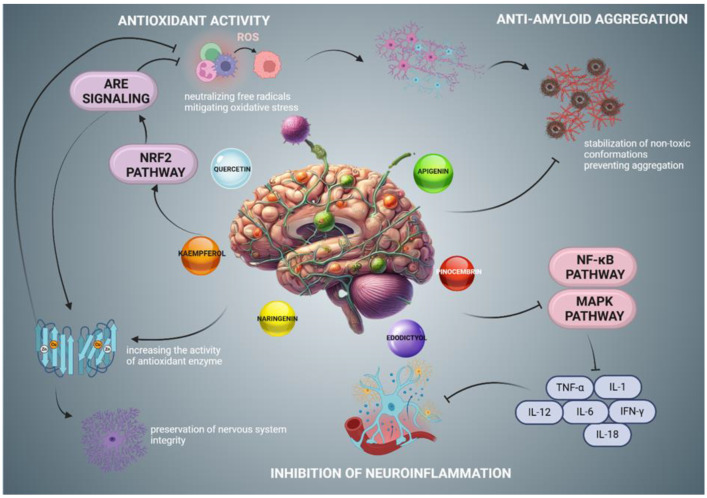
Neuroprotective effects of flavonoids. Flavonoids exhibit neuroprotective properties through multiple mechanisms. (1) Antioxidant activity: Flavonoids activate the Nrf2 (nuclear factor erythroid 2-related factor 2) pathway, enhancing antioxidant response element (ARE) signaling. This activation promotes the expression of antioxidant enzymes (such as glutathione and superoxide dismutase), which safeguard neurons from reactive oxygen species (ROS). (2) β-amyloid interaction: Flavonoids bind to β-amyloid, stabilizing non-toxic conformations and inhibiting aggregation, thereby reducing neurotoxic effects. (3) Anti-inflammatory effects: Flavonoids inhibit the NF-κB (nuclear factor kappa-light-chain-enhancer of activated B cells) and MAPK (mitogen-activated protein kinase) pathways, which are critical for the production of pro-inflammatory cytokines. This inhibition leads to decreased levels of TNF-α, IL-6, and other inflammatory cytokines, highlighting the role of flavonoids in modulating microglial activation and inflammation. Created in BioRender. Bijak, M. (2024) https://BioRender.com/w91a417 accessed on 27 December 2024.

Flavonoids exhibit a range of bioactive properties that underscore their significance in disease therapy and prevention. They function as potent antioxidants, neutralizing free radicals and mitigating oxidative stress, thereby protecting cells from damage. Their anti-inflammatory effects arise from the regulation of signaling pathways that govern the production of pro-inflammatory cytokines, which is critical in the context of inflammatory and neurodegenerative diseases [21,94,96]. Furthermore, flavonoids exhibit neuroprotective properties through three principal mechanisms essential for the preservation of nervous system integrity (Figure 1). First, their antioxidant activity involves the neutralization of free radicals, thereby protecting neuronal cells from oxidative stress. This process includes the elimination of reactive oxygen species, such as hydroxyl radicals (^•^OH) and superoxide anions (O_2^−^_^−^), as well as the activation of the Nrf2/ARE signaling pathway, which induces the synthesis of antioxidant enzymes, including glutathione and superoxide dismutase. This activation subsequently supports redox homeostasis within the neuronal cells [94,96]. Second, flavonoids inhibit the aggregation of β- amyloid, a pivotal pathogenic factor implicated in AD. Compounds such as quercetin and apigenin interact with β-amyloid oligomers, altering their conformation and preventing the formation of toxic aggregates, which may mitigate neurotoxicity and enhance cognitive function [21,97]. Third, flavonoids modulate inflammatory responses by reducing the production of pro-inflammatory cytokines, including TNF-α and IL-6, while simultaneously attenuating microglial activation, thus contributing to the reduction of neurodegenerative processes [98,99,100].

This intricate mechanism of action underscores the potential of flavonoids as critical components in therapeutic strategies targeting neurological disorders, emphasizing their role in promoting neuronal health and maintaining neurobiological homeostasis.

### 3.1. Quercetin

**Figure 2:**
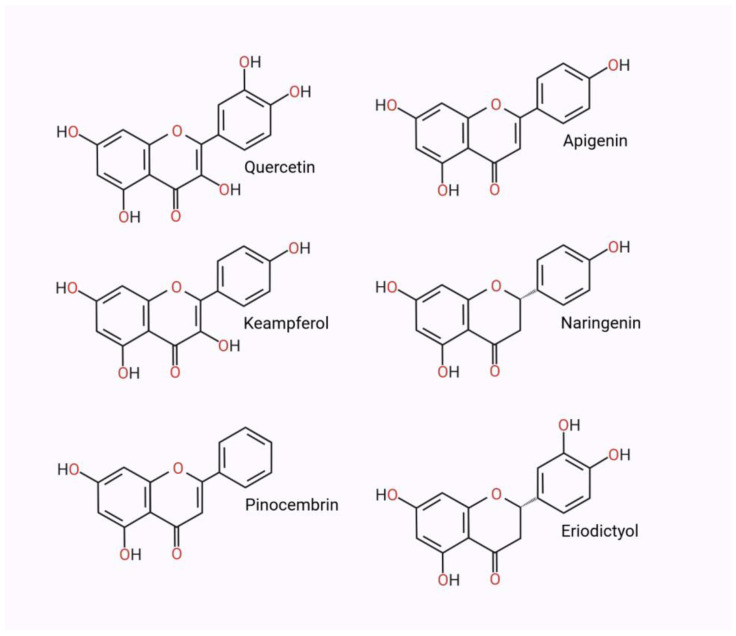
Chemical structures of flavonoids investigated for acetylcholinesterase inhibition and neuroprotective activity. Created in BioRender. Bijak, M. (2024) https://BioRender.com/o63e959 accessed on 27 December 2024.

Quercetin (3,3′,5,7,4′-pentahydroxyflavone) (Figure 2) is a flavonoid commonly found in foods such as apples, honey, raspberries, onions, red grapes, cherries, citrus fruits, and leafy green vegetables [22]. Quercetin aglycone is passively absorbed in the small intestine, while its glycosides are hydrolyzed by intestinal microbiota enzymes such as lactase-florin hydrolase and beta-glucosidase. Quercetin aglycone is metabolized following its primary, most important absorption into methylated, sulfated, and glucuronidated derivatives [23]. Although animal studies suggest that quercetin can cross the blood–brain barrier (BBB), its bioavailability remains low, typically at pico- to nanomolar concentrations, prompting ongoing research to enhance its bioavailability [21].

Numerous in vitro, in vivo, in silico, and clinical studies indicate quercetin’s neuroprotective properties in treating neurodegenerative diseases [24,25,26,27,28]. Quercetin exerts its effects through multiple mechanisms, with a notable focus on its role as an AChE inhibitor. In silico studies demonstrate its strong affinity for the active site of AChE, forming hydrogen bonds with key residues such as Ser200 and His440, resulting in reversible enzyme inhibition (IC50 = 4.59 ± 0.27 µM) [29]. Additionally, quercetin enhances cellular defense against oxidative stress by activating the Nrf2/ARE pathway, which upregulates antioxidant enzymes such as PON2 and SIRT1, contributing to redox homeostasis. Its anti-amyloidogenic effects, including the inhibition of β-amyloid fibril formation, are mediated by direct interaction with β-amyloid oligomers, stabilizing non-toxic conformations and reducing neurotoxicity. Quercetin also modulates neuroinflammatory markers by suppressing the overproduction of nitric oxide (NO) and reducing inducible nitric oxide synthase (iNOS) expression, further enhancing neuronal viability [30,31,32,33]. Additionally, quercetin inhibits β-amyloid fibril formation, enhances neurotrophin and brain-derived neurotrophic factor (BDNF) levels, and modulates inflammatory responses [34,35,36,37]. Furthermore, quercetin modulates kinase activity, reducing neuronal apoptosis and mitochondrial dysfunction, and scavenges free radicals, such as OH, H_2_O_2_, and O_2_^−^ [38,39].

Docking analyses suggest that quercetin’s structure allows for more effective enzyme inhibition compared to that of clinically used drugs, as evidenced by high binding energies and significant chemical interaction forces, such as pulling forces reaching up to 820 pN, positioning quercetin as one of the strongest AChE inhibitors among the flavonoids [40].

Alvarez-Berbel et al. investigated the mechanisms of AChE inhibition by both quercetin and apigenin. Their in vitro studies demonstrated that quercetin inhibited β-amyloid aggregation independently of AChE, while AChE significantly reduced the time required for Aβ fibrillation. In vivo analyses showed a dose-dependent inhibition of AChE by quercetin (IC50 = 40.7 µM), which resulted in increased ACh levels at the synapses, consequently improving cognitive functions in animal models. Improved cognitive functions in animal models were observed through increased synaptic availability of ACh due to the inhibition of AChE activity. For instance, quercetin supplementation led to enhanced performance in spatial memory and learning tasks by activating the Nrf2/ARE pathway, which upregulates antioxidant defenses, and by modulating neurotrophic factors such as BDNF (brain-derived neurotrophic factor) [41].

In a study by Liao et al., quercetin was shown to reversibly inhibit AChE activity in PC12 cells subjected to β-amyloid-induced oxidative stress. The formation of a stable complex was achieved through hydrogen bonding and van der Waals forces, with a binding constant of (5.52 ± 0.05) × 10^4^ L mol^−1^. Additionally, the study indicated that quercetin’s strong antioxidant properties mitigate neurotoxicity by neutralizing reactive oxygen species (ROS) such as hydroxyl radicals (•OH) and superoxide anions (O2•^−^). This antioxidative action prevents lipid peroxidation, preserves mitochondrial integrity, and reduces neuronal apoptosis. These mechanisms collectively enhance neuronal viability and contribute to improved memory and learning outcomes, as demonstrated in animal models of AD [29].

### 3.2. Apigenin

Apigenin (4′,5,7-trihydroxyflavone) (Figure 2) is another flavonoid found in nature as a dimer, biapigenin, primarily isolated from the buds and flowers of *Hypericum perforatum*. The most important dietary sources of apigenin include grapefruit, oranges, parsley, onions, wheat sprouts, certain spices, and chamomile [47]. Apigenin exhibits several beneficial biological activities, including anti-inflammatory, antioxidant, and neuroprotective properties [42]. Furthermore, apigenin has demonstrated anti-cancer effects by inducing apoptosis in cancer cells and inhibiting their proliferation [44]. In the study by Zhao et al., apigenin was shown to reduce oxidative stress and inflammation, potentially improving cognitive function and behavior in animal models of AD. Flavonoids, including apigenin, improve cognitive function and behavior through multifaceted actions, such as reducing β-amyloid-induced neurotoxicity, modulating inflammatory markers like TNF-α and IL-6, and preserving mitochondrial function. Apigenin, specifically, enhanced memory performance by suppressing oxidative stress and reducing microglial activation, which helped protect hippocampal neurons in these models [48]. Studies in animal models, such as rats and mice, confirmed that apigenin supplementation improved cognitive abilities and enhanced performance in memory tests, making it a promising candidate for further clinical investigations [45].

Apigenin is a flavonoid with notable inhibitory activity against AChE, primarily mediated by its ability to form hydrogen bonds with key amino acid residues at the enzyme’s active site, including Ser200 and His440. Hydrophobic interactions further stabilize apigenin within the active site, resulting in a dose-dependent reduction in AChE activity (IC50 = 40.7 µM). Apigenin also exhibits anti-inflammatory effects by modulating microglial activation and reducing levels of pro-inflammatory cytokines such as TNF-α and IL-6 [46]. This dual mechanism of cholinergic enhancement and neuroinflammation suppression contributes to its potential in ameliorating cognitive deficits and neurodegenerative processes. Furthermore, apigenin mitigates β-amyloid aggregation by directly interacting with Aβ fibrils, preventing toxic oligomer formation and reducing neurotoxicity [49].

In studies conducted by Dourado et al., apigenin was found to reduce AChE levels, leading to an increase in ACh concentrations at the synapses. This mechanism supports the potential of apigenin in enhancing cholinergic signaling, which is crucial for cognitive functions. Additionally, apigenin shows promise as a complementary therapy, potentially improving therapeutic outcomes when combined with other neurodegenerative disorders treatments [43].

### 3.3. Kaempferol

Kaempferol (3,4′,5,7-Tetrahydroxyflavone) (Figure 2), a flavonoid prevalent in various plant sources, exhibits a range of biological activities that warrant further investigation. Found in significant quantities in foods such as kale, spinach, tea, and grapes, kaempferol has been associated with multiple health benefits, including antioxidant, anti-inflammatory, and anti-cancer effects [51,53,54].

Kaempferol demonstrates strong AChE inhibitory activity through allosteric modulation of the enzyme’s conformation and interaction with the peripheral anionic site (PAS), obstructing ACh’s access to the catalytic active site [55]. Molecular docking studies reveal that kaempferol binds to residues at both the PAS and active site, enhancing its inhibitory potency. Additionally, kaempferol protects neuronal cells from β-amyloid cytotoxicity by inhibiting the aggregation and misfolding of Aβ42. Its neuroprotective effects are further supported by its ability to enhance mitochondrial integrity and modulate oxidative stress markers [53]. Kaempferol’s anti-inflammatory effects include the suppression of pro-inflammatory cytokine release and the upregulation of brain-derived neurotrophic factor (BDNF) via the ERK1/2-CREB signaling pathway, promoting synaptic plasticity and cognitive improvement. This flavonoid reduces Aβ misfolding and remodels toxic oligomers and fibrils into non-toxic aggregates, thereby decreasing Aβ deposition and showcasing its potential anti-AD disease effects [59]. Furthermore, as a natural antioxidant, kaempferol enhances the activity of key antioxidant enzymes such as superoxide dismutase (SOD) and glutathione (GSH), thus mitigating oxidative damage and lipid peroxidation. In d-galactose-induced cognitive impairment models, kaempferol improved oxidative stress markers and maintained mitochondrial integrity [60]. Additionally, kaempferol exhibits anti-inflammatory properties by modulating the release of pro-inflammatory factors, potentially alleviating neuroinflammation and associated memory impairments [50]. Kaempferol also demonstrates anti-apoptotic effects, protecting against neuronal cell death by inhibiting caspase activation and regulating pro- and anti-apoptotic protein levels [52]. It has been found to increase brain-derived neurotrophic factor (BDNF) expression via the ERK1/2-CREB signaling pathway, promoting neuroplasticity and learning [101].

Kaempferol is a flavonoid recognized for its potential as an AChE inhibitor, a characteristic demonstrated through both in vitro and in silico analyses. Molecular docking studies indicate that kaempferol can effectively bind to specific sites on AChE, including the peripheral anionic site. This binding potentially enhances its inhibitory action, likely through allosteric modulation of the enzyme’s conformation or by obstructing the access of ACh to the active site, functioning as a non-competitive inhibitor [58,61].

In vitro studies have demonstrated that kaempferol exhibits potent inhibition of AChE, with IC50 values indicating a significant capacity to reduce enzymatic activity. Structure–activity relationship analysis suggests that the presence and position of the hydroxyl groups in kaempferol may enhance its inhibitory effects on AChE [61].

Kaempferol has been evaluated in various animal models, demonstrating its significant potential for improving cognitive function and increasing ACh levels in the brain, which supports its therapeutic relevance for conditions like AD. In a study by Lin et al., kaempferol administration was shown to enhance memory performance and mitigate cognitive deficits in rodent models of neurodegenerative disorders [57]. Similarly, research by Kouhestani et al. indicated that kaempferol supplementation could lead to improvements in learning and memory capabilities in rats, further highlighting its neuroprotective effects [56]. Additionally, kaempferol’s ability to elevate ACh levels aligns with the cholinergic hypothesis of AD, suggesting that its mechanism of action could be valuable in managing the cognitive decline associated with the disease [61].

### 3.4. Naringenin

Naringenin (2*S*)-4′,5,7-Trihydroxyflavan-4-one) (Figure 2), a flavanone aglycone derived from naringin, is a naturally occurring bioflavonoid predominantly found in various citrus species. This compound has garnered significant scientific interest due to its diverse pharmacological activities and potential therapeutic applications. Empirical evidence indicates that both naringenin and its glycoside precursor, naringin, demonstrate potent inhibitory effects on AChE activity in both in vitro and in vivo models [62,63,64]. Molecular docking analyses have elucidated that both compounds possess significant potential as multi-target inhibitors of AChE, BChE, and β-site amyloid precursor protein cleaving enzyme 1 (BACE1), exhibiting binding affinities comparable to those of established cholinergic agonists. Furthermore, in vitro enzymatic assays have quantitatively assessed the inhibitory potency of naringin against AChE, revealing an IC50 of 0.45 mmol/L, characterized by a dose-dependent inhibition profile [102].

Naringenin inhibits AChE through interactions at both the active and peripheral anionic sites, exhibiting binding affinities comparable to those of established cholinergic agonists. In animal models, naringenin increases ACh levels at the synapses by reducing AChE activity, leading to enhanced cholinergic signaling and cognitive improvement. Additionally, naringenin mitigates β-amyloid-induced neurotoxicity by preserving mitochondrial function, reducing calcium dysregulation, and downregulating apoptosis-related proteins. Its antioxidant properties, mediated through the Nrf2/ARE pathway, further protect neurons from oxidative damage, while its anti-inflammatory effects are evident in the suppression of TNF-α and IL-6 expression [65,66]. Varshney and Garabadu demonstrated that naringenin significantly inhibits AChE, leading to elevated ACh levels in the synapses. In animal models, dementia was induced in male rats via intracerebroventricular administration of β-amyloid peptide (Aβ (1-42)). After 14 days of naringenin treatment, a notable improvement in cognitive impairment was observed. Naringenin also enhanced cholinergic function, as evidenced by the increased activity of choline acetyltransferase (ChAT) and ACh levels, while reducing AChE activity in the hippocampus, prefrontal cortex, and amygdala. Additionally, it improved mitochondrial function, integrity, and bioenergetics, which Aβ had compromised. Naringenin mitigated the elevated calcium levels in the mitochondria and the cytosol and counteracted increased apoptosis, as well as the negative changes in heme oxygenase-1 and mitochondrial calcium uniporter levels. Importantly, the A779 antagonist significantly reduced naringenin’s beneficial effects on behavioral, biochemical, and molecular changes induced by Aβ [67]. Similarly, in a study by Varshney et al., the effects of naringenin on cognitive impairment and neuroinflammation related to AD were examined using a rat model. Dementia was induced in male Wistar rats through intracerebroventricular administration of Aβ (1-42). Following 14 days of naringenin treatment, significant improvements in cognitive function were observed. Naringenin specifically enhanced cholinergic function by increasing levels of ACh and boosting the activity of ChAT, while concurrently decreasing AChE activity in the hippocampus and cortex. This modulation of AChE activity is critical, as it contributes to the preservation of ACh levels, thereby improving cholinergic transmission and cognitive performance. Additionally, the study revealed that naringenin reduced Aβ-induced oxidative stress and neuroinflammation, as evidenced by lowered levels of pro-inflammatory cytokines such as TNF-α and IL-6, alongside increased antioxidant enzyme activity. The compound also mitigated neuronal damage by decreasing the expression of apoptosis-related proteins [63].

These findings highlight the potential of naringenin as a promising therapeutic agent for cognitive disorders characterized by cholinergic dysfunction. The mechanism of action, primarily through AChE inhibition, indicates that naringenin may facilitate the restoration of ACh levels in the central nervous system, thereby enhancing cognitive function in affected individuals. Furthermore, the pleiotropic effects of naringenin are not limited to a single pathological condition but may have broader implications for various cognitive impairments associated with cholinergic dysregulation. Additionally, the multi-target inhibitory properties of naringenin suggest potential synergistic effects on cognitive function, addressing multiple facets of cholinergic regulation concurrently.

### 3.5. Pinocembrin

Pinocembrin ((2S),5,7-dihydroxyflavan-4-one) (Figure 2) is a natural flavonoid compound with the ability to cross the blood–brain barrier [70], exhibiting multifaceted pharmacological properties including antimicrobial, anti-inflammatory, antioxidant, and anticancer activities. This bioactive molecule has garnered significant attention in neuroscience research due to its neuroprotective effects, which are thought to be mediated through the modulation of autophagic activity and the inhibition of cellular apoptosis [71]. Pinocembrin modulates AChE activity indirectly by preserving cholinergic neurotransmission and enhancing synaptic plasticity. While direct evidence for AChE inhibition is limited, pinocembrin demonstrates significant neuroprotective effects by reducing neuroinflammation and oxidative stress. It attenuates microglial activation, suppresses pro-inflammatory signaling pathways such as NF-κB, and reduces the expression of IL-1β and TNF-α. Pinocembrin also protects the integrity of the neurovascular unit, improving cerebral perfusion and mitigating blood–brain barrier dysfunction, which are critical in AD pathology. These combined mechanisms contribute to enhanced cognitive function in animal models of neurodegeneration. In murine models of AD, pinocembrin attenuated cognitive deficits and inhibited neuronal degeneration [68]. Liu et al. investigated the neurotherapeutic efficacy of pinocembrin in ameliorating neurovascular dysfunction and cognitive deterioration in AD pathophysiology. The experimental data demonstrated that oral administration of pinocembrin exhibits significant neuroprotective properties in murine AD models through the downregulation of RAGE-mediated signal transduction cascades, consequently attenuating Aβ-induced neurotoxicity. The therapeutic mechanism is characterized by perturbations in neurovascular unit (NVU) homeostasis, enhancement of cerebral perfusion, and modulation of cholinergic neurotransmission, culminating in cognitive function maintenance. Furthermore, pinocembrin administration resulted in significantly attenuated neuroinflammatory cascades and oxidative stress markers, both crucial pathogenic mechanisms in AD progression. The compound demonstrated modulatory effects on the RAGE-dependent downstream signaling pathways, specifically the ERK–CREB–BDNF axis, suggesting its role in neuronal viability and NVU structural integrity maintenance [68].

The findings emphasize the compound’s potential to target multiple pathological cascades simultaneously, which is particularly relevant, given the complex etiology and progression of AD. The demonstrated oral bioavailability and significant therapeutic effects warrant additional preclinical and clinical investigations to elucidate its potential as a therapeutic intervention in AD treatment protocols. Similarly, it improved spatial learning and memory in diabetic mouse models [69]. While pinocembrin shows promise in alleviating neuroinflammation, its specific role in the inhibition of AChE remains to be fully elucidated, necessitating further investigations to fully determine its therapeutic potential in neurological diseases.

### 3.6. Eriodictyol

Eriodictyol ((2S)-3′,4′,5,7-Tetrahydroxyflavan-4-one) (Figure 2) is a structurally complex flavanone-class flavonoid predominantly found in citrus fruits and vegetables. Eriodictyol represents a promising multi-target therapeutic agent with demonstrated effects on neurological, cardiovascular, and cancer-related conditions. The binding interaction between eriodictyol and AChE has been experimentally demonstrated through an affinity-based protein assay utilizing AChE-conjugated magnetic beads coupled with LC-HRMS analysis. Specifically, eriodictyol 2′-O-glucoside/3′-O-glucoside showed selective binding to AChE, while eriodictyol 7-O-glucoside, despite its presence in the crude extract, did not demonstrate binding affinity. This position-specific binding selectivity suggests that the glucoside position plays a crucial role in the molecular recognition between eriodictyol derivatives and AChE [72].

Eriodictyol selectively binds to AChE, with position-specific glucoside derivatives influencing its inhibitory potential. In silico studies have identified eriodictyol 2′-O-glucoside as a high-affinity binder to AChE, forming stable interactions with key residues at the active site. Its neuroprotective effects are mediated through the activation of the Nrf2/ARE signaling pathway, enhancing endogenous antioxidant defenses such as glutathione peroxidase and superoxide dismutase. Additionally, eriodictyol reduces neuroinflammation by suppressing microglial activation and pro-inflammatory cytokine production. These actions, combined with its ability to prevent β-amyloid-induced oxidative damage, underscore its potential as a multi-target therapeutic agent. It protects against H_2_O_2_-induced neurotoxicity in PC12 cells and β-amyloid 25-35-induced neuronal death [73]. In cardiovascular health, eriodictyol exhibits protective effects through multiple pathways. It reduces myocardial ischemia/reperfusion injury by activating Janus kinase 2 (JAK2) [74] and suppressing pro-inflammatory and apoptotic responses. It also demonstrates anti-cancer potential through multiple mechanisms. It induces apoptosis and G2/M cell cycle arrest by inhibiting the mTOR/PI3K/Akt cascades [75], showing particular promise in lung carcinoma treatment. Its diverse molecular mechanisms, coupled with its selective binding properties and well-characterized metabolic pathway, make it an attractive candidate for further therapeutic development. The compound’s ability to simultaneously modulate multiple pathways while maintaining specific molecular interactions, such as its selective AChE binding, suggests potential applications in complex disease states involving multiple therapeutic targets.

## 4. Selection Criteria for Flavonoids and Future Perspectives

The flavonoids chosen for this review—quercetin, apigenin, kaempferol, naringenin, pinocembrin, and eriodictyol—were selected for their well-established ability to inhibit AChE, with extensive evidence from in silico, in vitro, and in vivo studies. These compounds not only demonstrate potent AChE inhibitory activity but also possess a wide range of additional neuroprotective properties, such as antioxidant, anti-inflammatory, and anti-amyloidogenic effects.

These flavonoids represent a diverse selection in terms of their structural diversity and mechanisms of action. By targeting multiple pathways associated with neurodegenerative diseases, including AD, they offer promising potential for improving cholinergic function and reducing neuroinflammation, oxidative stress, and amyloid toxicity.

The application of flavonoids as inhibitors of AChE activity presents considerable therapeutic potential; however, future research must prioritize enhancing their bioavailability and stability to facilitate an effective delivery across the blood–brain barrier. Strategies such as chemical modification, advanced formulation technologies, and the incorporation of excipients can significantly improve the pharmacokinetics of flavonoids, enabling sustained and targeted release within the central nervous system. Optimizing these parameters is critical for maximizing therapeutic efficacy and achieving the desired clinical outcomes. Moreover, the development of sophisticated delivery systems, including nanoparticles, liposomes, and hydrogels, represents a promising avenue for augmenting the therapeutic potential of flavonoids. These systems can enhance the solubility, stability, and biodistribution of flavonoids, facilitating targeted delivery to specific brain regions implicated in neurodegenerative processes. Additionally, employing surface modifications to improve cellular uptake and ensure the controlled release of flavonoids will enhance the precision of their pharmacological actions.

The safety profile of flavonoid interventions is a paramount concern, necessitating extensive in vivo and clinical studies. These investigations should encompass diverse cohorts, including individuals with varying genetic backgrounds, stages of disease, and comorbidities. Such inclusivity will yield a more comprehensive understanding of the effects and interactions of flavonoids, thereby paving the way for personalized therapeutic strategies.

Furthermore, flavonoids may serve as complementary agents to conventional therapies. Investigating the potential synergistic effects between flavonoids and established pharmacological treatments is essential for enhancing therapeutic regimens. Research should focus on elucidating how flavonoids can potentiate the efficacy of conventional drugs, mitigate their side effects, or reduce their requisite dosages. Identifying specific combinations that optimize efficacy while minimizing toxicity will be invaluable for the development of integrated therapeutic approaches.

## 5. Conclusions

Flavonoids have shown potential as natural AChE inhibitors in preliminary in vitro and in silico studies, suggesting their promise as complementary agents for neurodegenerative diseases, particularly AD. However, the translation of these findings into clinical practice requires further in vivo and clinical studies to assess their pharmacokinetics, bioavailability, and long-term efficacy in complex biological systems. The multifaceted effects of flavonoids—such as quercetin, apigenin, kaempferol, and naringenin—in modulating oxidative stress, reducing β-amyloid aggregation, and enhancing cholinergic signaling provide an integrated approach to neuroprotection. By effectively inhibiting AChE, these compounds help preserve ACh levels, which are essential for cognitive functions that are impaired in AD and related disorders. Flavonoids not only stabilize cholinergic neurotransmission but also mitigate neuroinflammation, thereby offering a dual therapeutic benefit. These characteristics have significant implications, suggesting that flavonoid-based interventions may enhance cognitive performance, slow the progression of neurodegeneration, and promote overall brain health. The ability of flavonoids to target multiple pathogenic pathways, such as AChE inhibition, oxidative stress reduction, and neuroinflammation modulation, suggests their potential as complementary treatments for neurodegenerative diseases. Unlike conventional treatments that primarily address symptoms, flavonoids offer a broader approach by targeting various aspects of disease progression. For instance, quercetin and kaempferol not only enhance cholinergic function but also mitigate β-amyloid aggregation and inflammation, supporting the notion of a more holistic treatment strategy in neuropharmacology. However, further research and clinical trials are essential to optimize the bioavailability of these flavonoids, assess their long-term efficacy, and elucidate their molecular mechanisms in human models. Such studies will be critical for supporting the integration of flavonoids into therapeutic protocols, ultimately enhancing treatment strategies for neurodegenerative diseases.

## Figures and Tables

**Table 1:** Neuroprotective mechanisms and biological actions of flavonoids with acetylcholinesterase-inhibitory activity.

Flavonoid	Source	Mechanism of Action on AChE	Neuroprotective Effect	Other Biological Actions	Mechanism of Action (Signaling Pathways)	Types of Studies	Bibliography
Quercetin	Apples Honey Raspberries Onions Red grapes Cherries Citrus fruits Leafy green vegetables	Forms hydrogen bonds at AChE’s active site. Dose dependent inhibition of AChE activity	Inhibits Aβ fibril formation. Enhances neurotrophin and BDNF levels. Reduces neuronal apoptosis, decreases mitochondrial dysfunction, and scavenges free radicals. Crosses blood–brain barrier (though with low bioavailability).	Anti-inflammatory properties. Antioxidant properties. Anti-cancer effects.	Activates Nrf2/ARE pathway. Stimulates antioxidant enzymes: PON2 SIRT1 Induces autophagy. Modulates inflammatory responses. Modulates kinase activity.	In silico, in vitro, in vivo.	[21,22,23,24,25,26,27,28,29,30,31,32,33,34,35,36,37,38,39,40,41]
Apigenin	Hypericum perforatum (buds and flowers) Grapefruit Oranges Parsley Onions Wheat sprouts Chamomile	Forms hydrogen bonds at AChE’s active site. Dose dependent inhibition of AChE activity.	Improves cognitive abilities.	Anti-inflammatory, antioxidant properties. Anti-cancer effects: Induces apoptosis in cancer cells. Inhibits cancer cell proliferation. Increases ACh concentrations at synapses.	Enhances cholinergic signaling. Decreases markers of inflammation. Reduces oxidative stress markers.	In silico, in vitro, in vivo.	[41,42,43,44,45,46,47,48,49]
Kaempferol	Kale Spinach Tea Grapes	Shows allosteric modulation of enzyme conformation and obstructs ACh access to active sites. Elevates ACh levels.	Protects neuronal cells from β-amyloid cytotoxicity by inhibiting the aggregation and misfolding of Aβ42. Remodels toxic oligomers and fibrils into non-toxic aggregates, thereby decreasing Aβ deposition. Improves cognitive abilities. Promotes neuroplasticity.	Antioxidant, anti-inflammatory, and anti-cancer effects. Inhibits caspase activation and Regulates pro- and anti-apoptotic protein levels. Maintains mitochondrial integrity.	Modulates release of pro-inflammatory factors. Increases BDNF expression. Regulates pro- and anti-apoptotic protein levels Enhances antioxidant enzyme activity: Superoxide dismutase (SOD) Glutathione (GSH).	In silico, in vitro, in vivo.	[50,51,52,53,54,55,56,57,58,59,60,61]
Naringenin	Various citrus species	Dose-dependent inhibition of AChE activity.	Improves cognitive abilities. Increases ACh levels in synapses. Reduces neuronal damage. Mitigates Aβ-induced effects. Preserves cholinergic transmission.	Improves mitochondrial: Function Integrity Bioenergetics. Mitigates elevated calcium levels in: Mitochondria Cytosol. Counteracts increased apoptosis. Reduces oxidative stress. Decreases expression of apoptosis-related proteins. Anti-inflammatory properties.	Increases activity of choline acetyltransferase (ChAT). Modulates mitochondrial calcium uniporter levels. Reduces neuroinflammation: Lowers TNF-α levels Decreases IL-6 levels Increases antioxidant enzyme activity.	In silico, in vitro, in vivo.	[62,63,64,65,66,67]
Pinocembrin	Propolis Honey Plants in the Rosaceae Family	Indirectly modulates AChE activity by preserving cholinergic neurotransmission and enhancing synaptic plasticity.	Crosses blood–brain barrier. Attenuates cognitive deficits. Inhibits neuronal degeneration. Enhances cerebral perfusion. Maintains cognitive function. Improves spatial learning and memory (in diabetic mouse models). Maintains neurovascular unit structural integrity.	Antimicrobial activity. Anti-inflammatory properties. Antioxidant properties. Anti-cancer activities. Modulates autophagic activity. Inhibits cellular apoptosis. Attenuates microglial activation. Attenuates Aβ-induced neurotoxicity.	Downregulates RAGE-mediated signal transduction cascades. Affects ERK-CREB-BDNF axis. Regulates neurovascular unit (NVU) homeostasis. Reduces neuroinflammatory cascades. Decreases oxidative stress markers.	In silico, in vitro, in vivo.	[68,69,70,71]
Eriodictyol	Citrus fruits Vegetables	Glucoside position-specific binding selectivity. Dose-dependent inhibition of AChE activity.	Protects against: H_2_O_2_-induced neurotoxicity. Aβ 25-35-induced neuronal death.	Cardiovascular protective effects: Reduces myocardial/ischemia/reperfusion injury. Suppresses pro-inflammatory response. Suppresses apoptotic response. Anti-cancer effects: Induces apoptosis. Causes G2/M cell cycle arrest.	Activation of: Nrf2/ARE signaling pathway. Janus kinase 2 (JAK2). Inhibits mTOR/PI3K/Akt cascades. Activates endogenous antioxidant defense mechanisms.	In silico, in vitro.	[72,73,74,75]

## Data Availability

Not applicable.
